# Still moving toward automation of the systematic review process: a summary of discussions at the third meeting of the International Collaboration for Automation of Systematic Reviews (ICASR)

**DOI:** 10.1186/s13643-019-0975-y

**Published:** 2019-02-20

**Authors:** Annette M. O’Connor, Guy Tsafnat, Stephen B. Gilbert, Kristina A. Thayer, Ian Shemilt, James Thomas, Paul Glasziou, Mary S. Wolfe

**Affiliations:** 1 College of Veterinary Medicine, Iowa State University, 1800 Christensen Drive, Ames, IA 50011 USA; 2 Evidentli Pty Ltd, Sydney, Australia; 3 College of Engineering, Iowa State University, Ames, IA 50011 USA; 4 US Environmental Protection Agency, Research Triangle Park, NC 27711 USA; 5 EPPI-Centre, University College London, London, WC1E 6BT UK; 6 Bond University, Robina, Queensland 4226 Australia; 7 National Institute of Environmental Health Sciences, Research Triangle Park, NC 27709 USA

**Keywords:** Systematic review, Evidence synthesis, Automation, Tools, Priority ranking, Data extraction, Data abstraction

## Abstract

The third meeting of the International Collaboration for Automation of Systematic Reviews (ICASR) was held 17–18 October 2017 in London, England. ICASR is an interdisciplinary group whose goal is to maximize the use of technology for conducting rapid, accurate, and efficient systematic reviews of scientific evidence. The group seeks to facilitate the development and widespread acceptance of automated techniques for systematic reviews. The meeting’s conclusion was that the most pressing needs at present are to develop approaches for validating currently available tools and to provide increased access to curated corpora that can be used for validation. To that end, ICASR’s short-term goals in 2018–2019 are to propose and publish protocols for key tasks in systematic reviews and to develop an approach for sharing curated corpora for validating the automation of the key tasks.

## Background

The International Collaboration for Automation of Systematic Reviews (ICASR) is an interdisciplinary group with a shared interest in maximizing the use of technology to aid the transfer of scientific research findings to practice and decision-making. The vast amount of available research data makes the task of combining the evidence overwhelming, and automation is viewed as an approach for ensuring maximum value is obtained from society’s investment in research. ICASR aims to develop the capability for conducting rapid, accurate, and efficient systematic reviews of scientific evidence. Previous ICASR meetings were held in September 2015 and in October 2016 [1].

## The third ICASR meeting: scope

The overall goals of the third ICASR meeting were to:

- Update participants on the current state of automation of systematic reviews, particularly data extraction technologies
- Foster coordination of efforts on automation tools and establishment of standards for automating the systematic review process
- Foster collaborations to address interoperability among automated tools

## Meeting agenda

The organizing committee invited approximately 50 participants, including users of summarized research, methodologists, and technologists. The 2-day meeting consisted of presentations by selected participants, large group discussions, and small group discussions on focused topics. The presentation topics varied by session: (1) new tools or available tools, (2) challenges for creating and adopting tools, and (3) potential solutions for barriers to the uptake and acceptance of automation.

## Common themes that emerged during the meeting

Three common themes emerged during the meeting:

- The available set of tools is growing, as are the available datasets
- Creating workflows with the available tools and future tools remains an unsolved issue
- Approaches to gaining acceptance for tools need to be more formalized

### The set of automated tools is growing

The discussions highlighted the increased availability of tools directly related to systematic reviews or repurposed for systematic reviews. Many communities are working on approaches for transferring published scientific findings to users that include clinicians, reviewers, guideline developers, software engineers, and computation linguistics. Many of the technical issues are similar and involve numerous subtasks not unique to systematic reviews, such as conversion of portable document format (PDF) files into raw text files and recognition of data presented in figures and tables. Other available tools are designed more specifically for systematic reviews, such as tools to recognize randomized controlled trials (RCTs), assess the risk of bias in RCTs, and identify and extract relevant studies from citation databases. The SR Toolbox website^1^ summarizes the tools available for systematic reviewers [2].

To highlight the diversity of tasks being developed and the groups working to develop tools, several presenters discussed their innovations. Those tools included:

- Metta, a metasearch engine used at the earliest stage of a systematic review [3], is designed for high-recall search and retrieval of records across five databases including PubMed, Embase, CINAHL, PsycINFO, and Cochrane Central Register of Controlled Trials. Several groups are simultaneously working on approaches for identifying RCTs using Metta, although Metta is not specific for RCTs. The group developing Metta also has an “RCT tagger” [4, 5].
- Cochrane Crowd is another approach for identifying RCTs that uses crowdsourcing. Approximately 1.5 million classifications of bibliographic records have been recorded, which represent the screening of more than 450,000 citations.

Most RCT identification tools use only the abstract and title for classification. Screening of full text represents a far more significant challenge, due to difficulties in processing PDF into text and the need to accommodate the larger variation of linguistic data in a full article. The meeting presentations, however, indicated that automated approaches for identifying RCTs with human study subjects are available and soon could be integrated into systematic review workflows. Discussion about automated approaches for recognizing other study designs was minimal.

Several groups are working on the creation of automated writing tools. The SEED (Systematic EvidEnce Disseminator) system [6], RevManHAL,^2^ and Trip Autosynthesis^3^^,^ 4 are examples of systematic review-specific tools. Reproducible research approaches that are agnostic to the application, such as knitr [7], are already available for publishing systematic reviews and can increase the pace of updating reviews.

Several groups also mentioned the availability of datasets for others to use. HAWC (Health Assessment Workspace Collaborative) is a content management tool used for manual data extraction.^5^ This open-source software is used by the US National Institute of Environmental Health Sciences (NIEHS), US Environmental Protection Agency (EPA), and others. Although over 3400 studies with extracted data are available within HAWC, no direct links from the extracted data to the PDF files are included. An annotated set of data from the Cochrane Schizophrenia Group is also available, which includes the location of the information in the PDF file.^6^

### Creating a workflow with the available tools

A major issue participants identified was creating a feasible workflow for combining the tools into an information pipeline. For some systematic review groups, developing a pipeline tailored explicitly for the group’s needs appears workable; for example, the Cochrane pipeline includes a tool for identifying RCTs. Such a tool, however, is of less utility to groups seeking to incorporate data from different study designs; for example, from experimental studies in animals, non-randomized trials, observational studies, or diagnostic test evaluations. Systematic review teams share a critical need to have greater interoperability among tools to cover more phases of the systematic review, although certain applications might need to be highly tailored to specific content areas.

The discussion about linking tools focused on two main themes: how to link tools and integrate them efficiently into the workflow. Regarding how to link tools, the debate continues about how best to create APIs (application programming interfaces) to enable users of different tools to exchange data. Just as most web browsers have APIs to display multiple image formats (such as GIF, JPG, PNG), systematic review tools ideally would support multiple formats of data exchange. Standardizing data formats, as the image community has, however, remains a need. The API discussion included a specified API system for systematic reviews called Piano, which follows ICASR’s Vienna Principles, facilitates interoperability among existing and de novo task-specific tools, and implements flexible, reproducible, and transparent workflows. The workflow was demonstrated with five tools that automated a systematic review on recurrence of heart failure after stent implementation. The participants emphasized the importance of ensuring that, as a first step, automation tools provide APIs, even without standardization, and that best practice is achieved for task-specific datasets so that they can be shared with tool developers. Many previously developed tools lack an articulated vision for integration into workflows, which wastes time and resources. Tools developed without consideration of workflow integration are more likely to wither after development or have their adoption delayed.

### Approaches to gaining acceptance for tools

A significant topic of discussion was how to encourage uptake of automated tools. Clearly, substantial barriers remain. Some barriers relate to general skepticism toward machine-assisted tasks. Such barriers might be corrected if the systematic review community had a broader understanding about what “machine-assisted” means. Increasing the knowledge of the various roles automation might play in systematic reviews likely would increase trust. For example, external stakeholders might believe the current vision is automated reviews devoid of valuable human control and input, that is, a general autonomous artificial intelligence system. That view, however, was neither represented nor sanctioned at the meeting. Therefore, improving the terminology associated with systematic review automation to reflect the goal more accurately is likely valuable. For example, the terms “machine-assisted,” “computer-assisted,” and “computer-supported” more accurately reflect the plausible path over the next decade.

Further, accounting for theories of diffusion or adoption of innovations [8, 9] was considered important. Knowledge of these theories would enable ICASR to target efforts more efficiently and lead to greater acceptance. Considerations include:

- *The need to document greater relative advantage*. The degree to which an innovation is perceived as better than the idea it supersedes will affect its adoption.
- *The need for compatibility.* A more significant degree of infrastructural and conceptual compatibility will facilitate adoption. The concept of compatibility mirrors the concept of integration into workflows.
- *The need for “trialability*.*”* Enabling users to experiment with innovation will promote adoption. Currently, tools are not “trialable” because of the effort involved in integrating them into a working review system. Adding a tool to a working system would disrupt the review, which would be problematic for reviews that already take too long to complete.
- *The need for observability*. Increasing visibility and clarity of steps and results in an innovation facilitates adoption.
- *The perceived complexity of the innovation*. Innovations perceived as easy to understand and use are adopted more readily.

Acceptance of tools is currently limited because most systematic review teams and reviewers are concerned about their validity and the community has not reached consensus on evaluation standards. Although prior ICASR meetings noted this need, little progress has occurred. A recently published paper found significant deficiencies in the approaches to reporting eligibility screening tools developed for systematic review [10].

Another major issue is the need for publicly available datasets and corpora that developers and adopters can use as benchmarks for ground-truthing tools. Although many participants had datasets appropriate for sharing, barriers exist. Barriers include licensing issues, obtaining credit for the work associated with developing the corpora, incorporating associated meta-data, and identification of the corpus. Those datasets have different forms. Most systematic review groups have annotated data that indicates a study characteristic, but the organization of the datasets differs; some groups simply classify the papers in the study according to particular characteristics and provide a citation with the classification. Other groups partner the classification with the PDF and annotated PDF or provide sentence- or phrase-level text annotations. Different approaches to reporting datasets are needed if the perceived value of making a dataset open access is to be realized. Further, the limited experience of the systematic review community with an open-access corpus, compared to communities like linguistics, makes unclear how widely the datasets will be used. Resources that are already developed are likely the best approach to ensuring the reproducibility and sustainability of dataset access with systems such as Meta-Share.^7^

## Revisiting the goals of ICASR 2017

At the conclusion of the second ICASR meeting, the following areas were identified as priorities:

- *Comprehension of required tasks/steps.* Whether this list is still needed is less clear because automation could serve a purpose in all steps of systematic reviews. Further, the definition of a task or step is unclear. For example, some groups are interested in developing tools that encompass an entire step in the systematic review process, while others are interested in subtasks. A list of priority tasks could actually stifle innovation.
- *Validation of tools.* This topic has risen to a higher priority. At earlier meetings, the focus was on needed tools. However, this view has shifted somewhat, given the growth of the number of groups working on systematic reviews along with the recognition that existing tools can be repurposed for their use. The need now is to validate the available tools, and protocols are seen as a way to achieve this validation.
- *Development of data extraction tools.* Progress has been made in many areas of data extraction, which refers to extracting the relevant content data from a paper’s methods and results and the meta-data about the paper, for example, authors and references. It seems likely that most tools being developed will involve computer assistance rather than complete automated detection.
- *Interoperability standards.* Interoperability remains an urgent need, and with each new tool that is not compatible with other systems, that need becomes more pressing.

## Conclusion and future goals

Based on the meeting’s discussion, the following are identified as ICASR goals:

- Develop an ICASR website, link to and from the SR Toolbox

That the systematic review community is aware of the available resources is critical so that duplication of effort is avoided. Therefore, one goal is to create a website for ICASR and increase awareness of the SR Toolbox. This site also might provide guidance on how to create corpora, enable access to corpora, or link to corpora for others to use.

- Develop guidelines for evaluating tools and reporting automated tasks for systematic reviews

A working group will be formed to develop two or three protocol publications.

## Abbreviations

APIs: Application programming interfaces
EPA: US Environmental Protection Agency
HAWC: Health Assessment Workspace Collaborative
ICASR: International Collaboration for Automation of Systematic Reviews
NIEHS: US National Institute of Environmental Health Sciences
PDF: Portable document format
RCTs: Randomized controlled trials
SEED: Systematic EvidEnce Disseminator

## Footnotes

1
http://systematicreviewtools.com/

2
http://schizophrenia.cochrane.org/revman-hal-v4

3
https://www.tripdatabase.com

4
https://blog.tripdatabase.com/2017/11/21/autosynthesis-timeline-to-release/

5
https://hawcproject.org

6 DOI: 10.13140/RG.2.2.28907.95529, Appendix: traceable extracted data from included studies of tardive dyskinesia reviews. Available at https://www.researchgate.net/publication/308698005_Appendix_Traceable_Extracted_Data_from_Included_Studies_of_Tardive_Dyskinesia_Reviews?channel=doi&linkId=57ebe1c508ae92a5dbd051c1&%2338;showFulltext=true

7
http://www.meta-share.org
